# Undernourishment *in utero* Primes Hepatic Steatosis in Adult Mice Offspring on an Obesogenic Diet; Involvement of Endoplasmic Reticulum Stress

**DOI:** 10.1038/srep16867

**Published:** 2015-11-19

**Authors:** Keiko Muramatsu-Kato, Hiroaki Itoh, Yukiko Kohmura-Kobayashi, Urmi J. Ferdous, Naoaki Tamura, Chizuko Yaguchi, Toshiyuki Uchida, Kazunao Suzuki, Koshi Hashimoto, Takayoshi Suganami, Yoshihiro Ogawa, Naohiro Kanayama

**Affiliations:** 1 Department of Obstetrics and Gynecology, Hamamatsu University School of Medicine, Hamamatsu 431-3192 Japan; 2 Department of Preemptive Medicine and Metabolism, Tokyo 113-8510, Japan; 3 Department of Organ Network and Metabolism, Tokyo 113-8510, Japan; 4 Department of Molecular Endocrinology and Metabolism, Graduate School of Medical and Dental Sciences, Tokyo Medical and Dental University, Tokyo 113-8510, Japan; 5 Japan Science and Technology Agency, PRESTO, Tokyo, Japan; 6 Japan Agency for Medical Research and Development, CREST, Tokyo, Japan

## Abstract

In order to investigate the possible involvement of endoplasmic reticulum (ER) stress in the developmental origins of hepatic steatosis associated with undernourishment *in utero*, we herein employed a fetal undernourishment mouse model by maternal caloric restriction in three cohorts; cohort 1) assessment of hepatic steatosis and the ER stress response at 9 weeks of age (wks) before a high fat diet (HFD), cohort 2) assessment of hepatic steatosis and the ER stress response on a HFD at 17 wks, cohort 3) assessment of hepatic steatosis and the ER stress response at 22 wks on a HFD after the alleviation of ER stress with a chemical chaperone, tauroursodeoxycholic acid (TUDCA), from 17 wks to 22 wks. Undernourishment *in utero* significantly deteriorated hepatic steatosis and led to the significant integration of the ER stress response on a HFD at 17 wks. The alleviation of ER stress by the TUDCA treatment significantly improved the parameters of hepatic steatosis in pups with undernourishment *in utero*, but not in those with normal nourishment *in utero* at 22 wks. These results suggest the pivotal involvement of the integration of ER stress in the developmental origins of hepatic steatosis in association with undernourishment *in utero*.

Non-alcoholic fatty liver disease (NAFLD) is considered to be a hepatic manifestation of metabolic syndrome and the leading cause of hepatic dysfunction. The prevalence of NAFLD in the general population was previously estimated to be 20–30% in Western Countries and 5–18% in developing countries, such as Asia^1^. However, the rate of increases in the prevalence of NAFLD was shown to be markedly higher in developing countries than in developed countries^2^,^3^. The prevalence of NAFLD in China and Japan has nearly doubled in the last 10–15 years^2^, and has been attributed to the widespread availability of obesogenic cheap energy-dense foods. Nevertheless, the natural history of NAFLD remains unclear. Nobili *et al.* suggested the importance of the contribution of the programming hypothesis of pro-steatotic conditions presumably caused by undernourishment or overnourishment *in utero*^4^. Developing countries have been undergoing rapid economic improvements over the past few decades, and a generation that was exposed to a low nutritional environment during fetal life due to maternal poverty and/or political turmoil has now shifted to a life of an obesogenic diet. Recent human^5^,^6^ and animal studies^7^,^8^,^9^ revealed that undernourishment *in utero* was causatively associated with the risk of NAFLD in later life. Therefore, it is plausible that not only the rapid shift to obesogenic energy-dense foods, but also exposure to undernourishment *in utero*, may underlie the marked increase in the rate of adult NAFLD in developing countries. However, how undernourishment *in utero* programs adult NAFLD has not yet been fully elucidated; therefore, effective preventive strategies against its prevalence, specifically in developing countries, have not been established.

The endoplasmic reticulum (ER) is the major site in cells for protein folding as well as trafficking and the critical site of the metabolism, secretion, and homeostasis of proteins, lipids, and glucose^10^,^11^. The ER is considered to play important roles in the regulation of lipid droplet number, composition, and size and/or in lipogenesis as well as lipolysis; however, its involvement still remains unclear^11^,^12^. The ER plays a vital role in maintaining cellular and organismic metabolic homeostasis under normal physiological fluctuations in nutrients and conditions of excess^10^,^11^. However, a failure in the adaptive capacity of the ER, ER stress, has been shown to activate the unfolded protein response (UPR), which intersects with many different inflammatory and stress signaling pathways^13^. Thus, the ER stress response represents an evolutionary bottleneck that leads to common chronic diseases, including hepatic steatosis^14^,^15^, as well as a valuable target area for their prevention and treatment^15^. Experimental studies^14^,^16^ and evidence obtained from humans^17^,^18^ revealed the important causative involvement of ER stress in the development of hepatic steatosis.

In the present study, we hypothesized that undernourishment *in utero* programs the up-regulation of ER stress in the adult liver on an obesogenic high fat diet (HFD) and causes the deterioration of hepatic steatosis. To prove the hypothesis, we employed a fetal undernourishment mouse model by maternal caloric restriction, which we established previously^19^,^20^,^21^,^22^,^23^, to three cohorts as illustrated in Fig. 1; cohort 1) assessment of hepatic steatosis and the ER stress response at 9 weeks of age (wks) before a HFD, cohort 2) assessment of hepatic steatosis and the ER stress response on a HFD at 17 wks, cohort 3) assessment of hepatic steatosis and the ER stress response at 22 wks on a HFD after the alleviation of ER stress by the chemical chaperone, tauroursodeoxycholic acid (TUDCA) from 17 wks to 22 wks.

## Materials and Methods

### Animal models

Pregnant C57Bl/6 mice (n = 20 [cohorts 1 and 2], n = 40 [cohort 3]) were purchased at 7.5 days *post coitum* (dpc) from Japan SLC, Inc. (Hamamatsu, Japan) and housed individually with free access to water during 12-h/12-h light/dark cycles under a regular chow diet (formula number D06121301, Research Diets Inc., New Brunswick, NJ). Dams were divided into two groups at 11.5 dpc. One group was fed the powdered regular chow *ad libitum* (AD, n = 10 [cohorts 1 and 2], n = 20 [cohort 3]). The maternal caloric intake of the other group was restricted to 60%, i.e. 40% reduction, (CR; n = 10 [cohorts 1 and 2], n = 20 [cohort 3]) of the *ad libitum* feeding dams (AD), as previously described^19^,^20^,^21^,^22^,^23^, between 11.5 dpc and 17.5 dpc (Supplemental Figure S1). Caloric restriction of 60%, i.e. 40% reduction, was adopted in the present study because liver weight and the liver weight/body weight ratio in the adult offspring of CR dams were both found to be significantly higher than those in AD dams in pilot studies of caloric restriction of 70%, 65%, 60%, and 55% (Supplemental Table S1). Pups of maternal caloric restriction of 60% did not show significant changes in mean body weight or mean caloric intake (Supplemental Table S2) with all significant increase in liver weight (Supplemental Table S1), suggesting a possibility of lipid redistribution from adipose tissues and ectopic deposition in the liver in this animal model.

**Figure 1:**
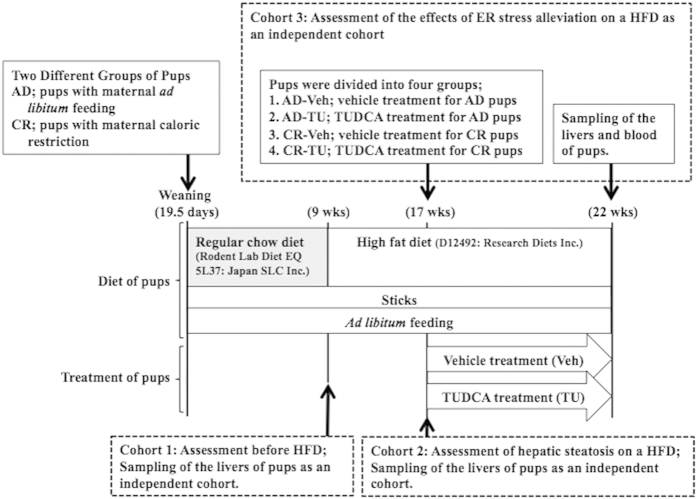
Schematic illustration of experimental procedures. Three cohort studies were carried out until 9 wks (cohort 1), 17 wks (cohort 2), and 22 wks (cohort 3). Procedure for the caloric restriction of dams was described in Supplemental Figure S1.

AD and CR dams were both fed *ad libitum* after delivery. All pups were cross fostered by dams fed *ad libitum* at 1.5 days of age, only male pups were selected by identifying black dots at the base of their tails, and the number of pups was adjusted to 8 per litter. Only male pups were used in subsequent experiments because obesity and its associated metabolic disorders on a HFD were previously reported to be more prominent in males than in females in this animal model^23^. A regular chow diet (Rodent Lab Diet EQ 5L37, Japan SLC, Inc., Hamamatsu, Japan) was supplied to all of the offspring after weaning until 9 wks (Fig. 1). Each cohort study was carried out as an independent study.

In cohorts 2 and 3, a HFD containing 60% lipids (formula number D12492, Research Diets Inc.) was supplied to all of the pups from 9 to 17 wks and from 9 to 22 wks, respectively, for the purpose of mimicking a modern obesogenic diet, as previously described^19^,^23^,^24^ (Fig. 1).

All experimental procedures were conducted in accordance with the standards of humane animal care by the criteria outlined in the “Guide for the Care and Use of Laboratory Animals” prepared by the National Academy of Sciences and published by the National Institutes of Health (NIH publication 86–23 revised 1985) and approved by the Animal Research Committee, Hamamatsu University School of Medicine (H20-014).

### ER stress alleviation by the TUDCA treatment under a HFD

In cohort 3, a HFD was supplied between 9 and 22 wks to AD and CR pups, as described above. At 17 wks, AD and CR pups were both randomly selected as a pup per litter and divided into two groups for Vehicle (Veh) or the TUDCA (TU; Merck Japan Ltd., Tokyo, Japan) treatment, resulting in four study groups being prepared, i.e. AD-Veh (n = 8), AD-TU (n = 10), CR-Veh (n = 7), and CR-TU (n = 10) groups (Fig. 1). TUDCA (TU), dissolved in distilled water and vehicle distilled water (Veh) were per orally administrated to the pups (TU: 0.5 g/kg/day) for five consecutive days a week from 17 to 22 wks until sampling (Fig. 1). Stainless needles (Product number; KN-348: Natsume. Ltd., Tokyo, Japan) were used for oral administration. At 22 wks, all pups were killed and the liver tissues as well as blood specimens were collected to assess hepatic steatosis and the ER stress response under a HFD (Fig. 1).

### Blood and tissue sampling

Sampling points are illustrated in Fig. 1. Two trained technicians blinded to the study systematically decapitated each pup under *ad libitum* feeding and immediately measured blood glucose levels with ACC-CHEK Compact Plus (Roche Diagnostics Japan, Tokyo, Japan). The remaining blood samples were collected with heparin-coated glass tubes and centrifuged at 1200 *g* for 15 min at 4 °C. The plasma thus obtained was aliquoted and stored at −30 °C until assayed. The whole liver was dissected and weighed. Some of the liver tissue was fixed in 10% formaldehyde and embedded in paraffin for a morphological analysis. The remaining tissue was snap frozen using liquid nitrogen in blocks and stored at −80 °C for mRNA or protein extraction.

### Measurement of lipids

Total lipids in the liver were extracted with ice-cold 2:1 (vol/vol) chloroform/methanol. Triglyceride (TG) concentrations were measured using enzymatic assay kits (Sekisui Medical Co. Ltd, Tokyo, Japan). Plasma lipoproteins were analyzed by an HPLC system at Skylight Biotec (Akita, Japan) according to the procedure described by Usui *et al.*^25^. The size of lipoprotein particles was determined based on individual elution times that corresponded to peaks on the chromatographic pattern of cholesterol fractions according to the procedure described by Okazaki *et al.*^26^.

### Histological assessment of hepatic steatosis

The liver tissue blocks embedded in paraffin were cut into 3-μm-thick sections. Hematoxylin and Eosin (HE) staining and Picrosirius Red staining (Cosmo Bio Co., Ltd, Tokyo, Japan) were carried out. Hepatic steatosis (grade) and cell ballooning were assessed and scored as 0 ~ 3 and 0 ~ 2, respectively, according to the procedures described by Kleiner *et al.*^27^. Hydroxyproline content was measured using a commercially available kit.

A macrophage-specific F4/80 rat monoclonal antibody (MCA497GA, AbD Serotec, Kidlington, UK) or CD45 rabbit polyclonal antibody (Proteintech Group, Inc., Chicago, IL, USA) was applied to the sections. Detection was performed with a polymer detection kit (ChemMate EnVision^TM^; Dako Japan, Tokyo, Japan) according to the manufacturer’s instructions, followed by a reaction with 3,3′-diaminobenzidine and counterstaining with hematoxylin. Eight separate images of a high-power field (HPF; ×400) were randomly separated and digitally captured, and the numbers of positive cells were then counted and assessed as numbers per HPF.

### Western blot

Twenty micrograms of protein from the liver tissues was loaded onto the SDS-Page gel and probed with primary antibodies against fatty acid synthase (FAS), sterol regulatory element-binding protein-1 (SREBP1), phospho-inositol-requiring enzyme 1α (p-IRE1α), inositol-requiring enzyme 1α (IRE1α), phospho-eukaryotic initiation factor 2α (p-eIF2α), eukaryotic initiation factor 2α (eIF2α), C/EBP-homologous protein (CHOP), glucose-regulated protein 78 (GRP78), or β-actin. Rabbit polyclonal antibodies against SREBP1 and β-actin were obtained from Abnova, Taipei, Taiwan and BioVision, Milpitas, CA, USA, respectively. Rabbit polyclonal antibody against p-IRE1α was obtained from Abcam Japan, Tokyo, Japan. All of the other primary antibodies (rabbit monoclonal) were obtained from Cell Signaling Technology Inc., Boston, MA, USA. After washing, the membrane was incubated with a HRP-conjugated goat polyclonal second antibody (R&D Systems, Minneapolis, MN, USA; 1:1,000). The intensities of the immunoblots were quantified using ImageJ (version 1.48). In case of analysis of 11 specimens (cohort 1), single blot was used. In case of analysis of 19 specimens (cohort 2), two blots were assessed by comparison with one identical specimen (duplicate, total two) as an internal positive control, as previously described^28^,^29^. In case of analysis of 24 specimens (cohort 3), four blots were similarly assessed by comparison with two identical specimens (in duplicate, total four), as internal positive controls^28^,^29^.

### Measurement of transaminase and insulin

Alanine transaminase (ALT) and aspartate transaminase (AST) levels were measured using FUJI DRI-CHEM 3500 (FUJIFILM Holdings Co., Tokyo, Japan). Insulin levels were measured using a commercially available kit.

### Quantitative RT-PCR analysis

The gene expression levels of unspliced form X-box binding protein 1 (XBP1u; inactive form), spliced from X-box binding protein 1 (XBP1s; active form), Tumor necrosis factor-α (TNF-α), and TNF receptor-associated factor 1 (TRAF1) were determined by quantitative RT-PCR using the High Capacity RNA to cDNA Master Mix (Applied Biosystems, Foster City, CA) and SYBR Green PCR Master Mix (Applied Biosystems), according to the manufacturer’s recommendations. The expression of 18S ribosomal RNA was used as an internal control. The primers used were XBP1u; forward; GGATCCTGACGAGGTTCCAG, reverse; GCAGAGGTGCACATAGTCTGA, XBP1s; forward; GAAAGAAAGCCCGGATGAGC, reverse; ACCTGCTGCGGACTCA, TNF-α; forward; ACCCTCACACTCAGATCATCTTC, reverse; TGGTGGTTTGCTACGACGT, TRAF1; forward; GGGAGCCCACAATCCATGCA, reverse; TCGCTTCCACAGCTGCCTGA, and 18S ribosomal RNA; forward; GGGAGCCTGAGAAACGGC, reverse; GGGTCGGGAGTGGGTAATTTT.

### Statistical analysis

Data are expressed as means ± SDs. The significance of differences between two mean values was assessed using the Student’s *t*-test or Mann-Whitney U test, as appropriate. The significance of differences among four mean values was assessed with the Steel-Dwass test. A *p* value of less than 0.05 was regarded as significant.

## Results

### Undernourishment *in utero* neither induced hepatic steatosis nor integrated the ER stress response in the liver at 9 wks before a HFD (cohort 1)

**Figure 2:**
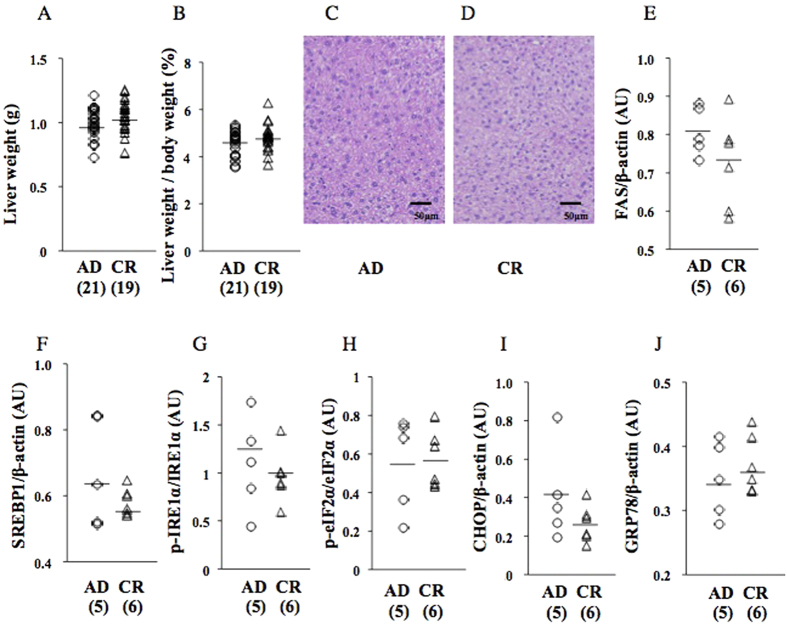
Effects of undernourishment *in utero* on parameters of hepatic steatosis (**A**,**B**) or the ER stress response at 9 wks before a HFD (cohort 1). HE staining (**C**,**D**). Western blot analysis of FAS (**E**), SREBP1 (**F**), p-IRE1α (**G**), p-eIF2α (**H**), CHOP (**I**), and GRP78 (**J**). A pup per litter was randomly selected for the experiments (**C**–**J**). AU; arbitrary unit. All bands of Western blot analysis (**E**–**J**) were shown in Supplemental Figure S2.

Liver weight and the liver weight/body weight ratio in CR pups were similar to those in AD pups (Fig. 2A,B). HE staining showed no apparent steatosis in AD (Fig. 2C) or CR pups (Fig. 2D) at 9 wks before a HFD. The protein expression levels of FAS and SREBP1 in CR pups were similar to those in AD pups (Fig. 2E,F). The number of F4/80-positive hepatic macrophages was in CR pups (10.5 ± 4.0 cells/HPF, n = 6) was similar to that in AD pups (11.0 ± 6.2 cells/HPF, n = 5).

The protein expression levels of p-IRE1α (Fig. 2G), p-eIF2α (Fig. 2H), CHOP (Fig. 2I), and GRP78 (Fig. 2J) in CR pups were similar to those in AD pups.

### Undernourishment *in utero* significantly deteriorated hepatic steatosis and hepatic inflammatory reactions, and significantly integrated the ER stress response at 17 wks on a HFD (cohort 2)

**Figure 3:**
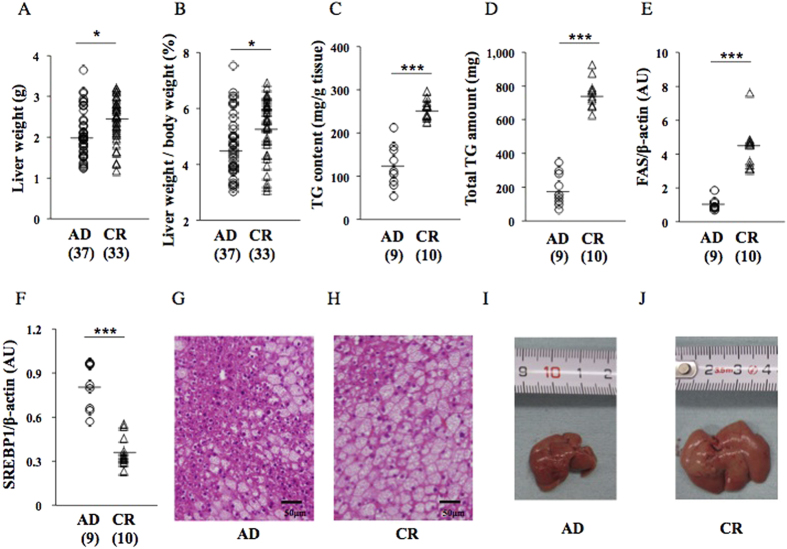
Effects of undernourishment *in utero* on parameters of hepatic steatosis (**A**–**D**) at 17 wks on a HFD (cohort 2). Western blot analysis of FAS (**E**) and SREBP1 (**F**). HE staining and gross appearance of the liver of AD (**G**,**I**)) and CR pups (**H**,**J**). A pup per litter was randomly selected for the experiments (**C**–**J**). *P < 0.05. ***P < 0.001. AU; arbitrary unit. All bands of Western blot analysis (**E**,**F**) were shown in Supplemental Figure S2.

**Figure 4:**
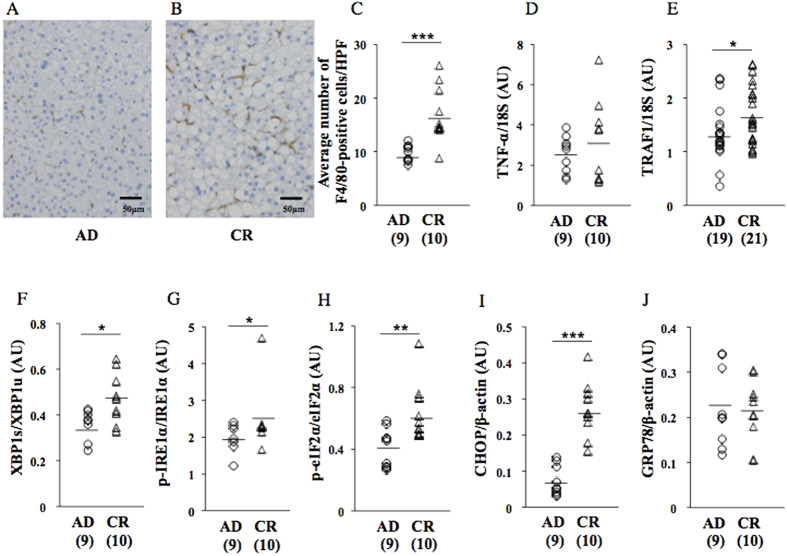
Effects of undernourishment on the average number of hepatic macrophages (**A**–**C**) and ER stress response at 17 wks on a HFD (cohort 2). Gene expression of TNF-α (**D**) and TRAF1 (**E**) and gene splicing of XBP1u (XBP1s; F) by a quantitative RT-PCR analysis. Western blot analysis of p-IRE1α (**G**), p-eIF2α (**H**), CHOP (**I**), and GRP78, an endogenous chaperone protein that inhibits hepatic lipogenesis (**J**). A pup per litter was randomly selected for the experiments (**A**–**J**). *P < 0.05. **P < 0.01. ***P < 0.001. AU; arbitrary unit. All bands of Western blot analysis (**G**–**J**) were shown in Supplemental Figure S2.

At 17 wks on a HFD, liver weight, the liver weight/body weight ratio, hepatic TG content, and hepatic total TG amount were significantly higher in CR pups than in AD pups (Fig. 3A–D). HE staining (Fig. 3G,H) and gross appearance (Fig. 3I,J) showed that the deterioration of hepatic steatosis was greater in CR pups than in AD pups. FAS protein expression levels were significantly higher in CR pups than in AD pups (Fig. 3E), while SREBP1 protein expression levels were significantly lower in CR pups than in AD pups (Fig. 3F). The number of F4/80-positive hepatic macrophages was significantly higher in CR pups than in AD pups (Fig. 4A–C). TRAF1 gene expression levels were significantly higher in CR pups than in AD pups (Fig. 4E), while TNF-α levels were slightly higher in CR pups than in AD pups (Fig. 4D).

The integration of the ER stress response in CR pups relative to AD pups was indicated by a significant increase in the ratio of XBP1s/XBP1u gene expression (Fig. 4F) as well as the protein expression of p-IRE1α (Fig. 4G), p-eIF2α (Fig. 4H), and CHOP (Fig. 4I), but not GRP78, an endogenous chaperone protein that inhibits hepatic lipogenesis (Fig. 4J).

### The amelioration of ER stress by the TUDCA treatment significantly alleviated hepatic steatosis, augmentation of hepatic macrophage numbers, the ER stress response, and plasma lipid profiles in pups with undernourishment *in utero*, but not in normal control pups at 22 wks on a HFD (cohort 3)

**Figure 5:**
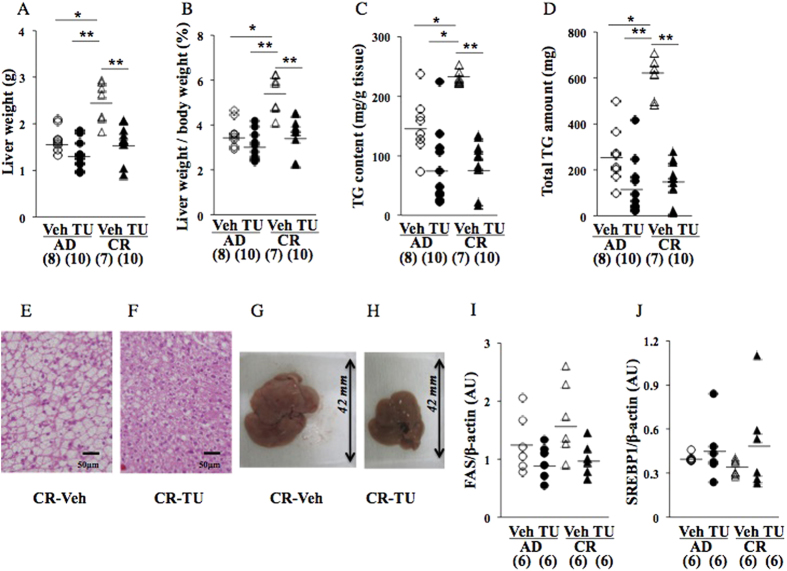
Effects of the TUDCA treatment on parameters of hepatic steatosis (**A–D**) at 22 wks on a HFD (cohort 3). HE staining (**E**,**F**) and gross appearance (**G**,**H**) of the liver. Western blot analysis of FAS (**I**) and SREBP1 (**J**). *P < 0.05. **P < 0.01. AU; arbitrary unit. All bands of Western blot analysis (**I**,**J**) were shown in Supplemental Figure S2.

The TUDCA treatment (17–22 wks) significantly decreased liver weight (Fig. 5A), the liver weight/body weight ratio (Fig. 5B), hepatic TG content (Fig. 5C), and hepatic total TG amount (Fig. 5D) in CR pups (CR-Veh v.s. CR-TU), but not in AD pups (AD-Veh v.s. AD-TU; Fig. 5A–D). HE staining (Fig. 5E,F) and gross appearance (Fig. 5G,H) also revealed that the TUDCA treatment improved steatosis in CR pups (CR-Veh v.s. CR-TU). The TUDCA treatment slightly decreased the protein expression levels of FAS (Fig. 5I), but not those of SREBP1 (Fig. 5J).

**Figure 6:**
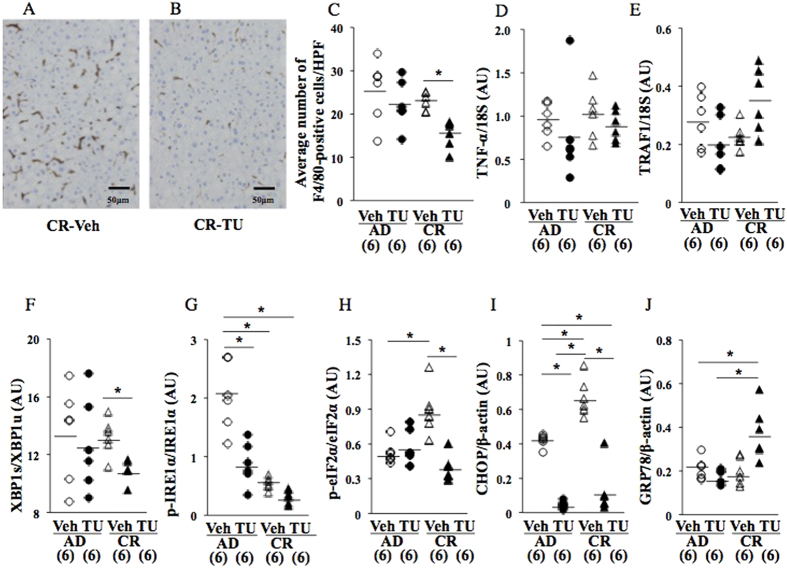
Effects of the TUDCA treatment on the average number of hepatic macrophages (**A**–**C**) and ER stress response at 22 wks on a HFD (cohort 3). The gene expression of TNF-α (**D**) and TRAF1 (**E**) and gene splicing of XBP1u (XBP1s; **F**) by a quantitative RT-PCR analysis. Western blot analysis of p-IRE1α (**G**), p-eIF2α (**H**), CHOP (**I**), and GRP78, an endogenous chaperone protein that inhibits hepatic lipogenesis (**J**). *P < 0.05. AU; arbitrary unit. All bands of Western blot analysis (**G**–**J**) were shown in Supplemental Figure 2S.

The TUDCA treatment significantly reduced the number of F4/80-positive hepatic macrophages in CR pups (Fig. 6A–C; CR-Veh v.s. CR-TU), but not in AD pups. The TUDCA treatment also slightly decreased the gene expression levels of TNF-α (Fig. 6D), but not those of TRAF1 (Fig. 6E).

The alleviation of the ER stress response in CR pups by the TUDCA treatment was indicated by a significant decrease in the gene splicing of XBP1u (XBP1s; Fig. 6F) (CR-Veh v.s. CR-TU), and in the protein expression of p-eIF2α (Fig. 6H) and CHOP (Fig. 6I). TUDCA treatment slightly decreased p-IRE1α protein expression in CR pups (Fig. 6G). The TUDCA treatment slightly increased the protein expression levels of GRP78, an endogenous chaperone protein that inhibits hepatic lipogenesis (Fig. 6J; CR-Veh v.s. CR-TU).

The TUDCA treatment significantly decreased the plasma levels of total cholesterol, LDL cholesterol, HDL cholesterol, and small dense LDL cholesterol in CR pups (CR-Veh v.s. CR-TU), but not in AD pups (Table 1A), but did not cause significant changes in the serum levels of chylomicron cholesterol, VLDL cholesterol, total triglycerides, glucose, or insulin (Table 1A).

Note; all bands of Western blots analysis were shown in Supplemental Figure S2. Results of Western blot analysis in cohort 2 and cohort 3 were summarized in Supplemental Table S3.

## Discussion

We herein demonstrated, using this mouse animal model, that undernourishment *in utero* programmed the exacerbation of fatty liver on an obesogenic HFD (Fig. 3). The parameters of fatty liver, i.e. liver weight, the liver weight/body weight ratio, TG content, and total TG amount, were significantly elevated by exposure to undernourishment *in utero* (AD v.s. CR; Fig. 3A–D). However, the scores of hepatic steatosis, and cell ballooning in CR pups were similar to those in AD pups (Table 1B). Hepatic fibrosis assessed by Picrosirius Red staining and hydroxyproline content in CR pups were similar to those in AD pups (Supplemental Figure S3 and Table 1C). These results indicated that undernourishment *in utero* appeared to cause a simple increase in hepatic total TG storage in this animal model. A significant elevation in hepatic FAS protein expression levels, a rate-limiting enzyme in hepatic fatty acid synthesis, (Fig. 3E) supported this speculation. A significant decrease in SREBP1 protein expression levels was observed in the CR pups of this animal model (Fig. 3F), which we currently have no clear explanation for. Since we did not prove direct involvement of ER stress integration in the regulation of lipid synthesis in the present study, it is necessary to investigate the epigenetic modification of these critical substances, i.e. SREBP1 and FAS, to clarify entire pathophysiological association between the exacerbation of hepatic steatosis and undernourishment *in utero*.

Previous studies demonstrated the important involvement of chronic inflammation in the development of hepatic steatosis^10^,^30^,^31^. In the present study, a significant elevation in the average number of F4/80-positive hepatic macrophages was observed in CR pups at 17 wks (Fig. 4A–C) concomitant with a slight increase in TNF–α gene expression levels (Fig. 4D) as well as the significant augmentation of TRAF1 gene expression (Fig. 4E). Therefore, this animal model was considered to have appropriately mimicked the risk of hepatic steatosis caused by fetal undernourishment in humans^5^,^6^ and coincided with the findings of other animal studies^7^,^8^,^9^.

The endoplasmic reticulum (ER) is the major site in cells for protein folding as well as trafficking^10^,^11^ and is also the site of triglyceride formation, particularly in liver cells^32^. Experimental models^11^,^15^,^16^ and evidence obtained from humans^17^,^33^,^34^ revealed that the activation of specific UPR pathways with ER stress was causatively associated with the development of hepatic steatosis, especially on an obesogenic diet. However, to the best of our knowledge, it currently remains unclear whether undernourishment *in utero* primes ER stress in the adult liver during the process of fat accumulation.

In the present study, undernourishment *in utero* significantly augmented the protein expression levels of p-IRE1α, a phospho-inositol-requiring enzyme 1α, with endonuclease activity^35^ (Fig. 4G), and the gene expression levels of XBP1s produced by mRNA splicing^36^ (Fig. 4F). Regarding the PERK pathway^37^, undernourishment *in utero* significantly augmented the protein expression levels of p-eIF2α (Fig. 4H) and CHOP (Fig. 4I), but not those of GRP78 (Fig. 4J), an endogenous chaperone protein, which is typically induced by UPR and inhibits hepatic lipogenesis to maintain lipid homeostasis^11^,^38^. Therefore, undernourishment *in utero* activated the UPR pathways of lipogenesis (Fig. 4F–I), but did not affect the protein expression levels of GRP78, a suppressor of lipogenesis^11^,^38^ (Fig. 4J), suggesting that it may induce a kind of predisposition to hepatic fat deposition on an obesogenic HFD, presumably by differently programming hepatic GRP78 protein expression from other UPR pathways at 17 wks (cohort 2).

At 22 wks (cohort 3), significant deteriorations were still observed in all the parameters of hepatic steatosis in the pups with undernourishment *in utero* (Fig. 5A–D; AD-Veh v.s. CR-Veh). In order to elucidate the involvement of ER stress in the developmental origins of the risk of hepatic steatosis in more detail, we ameliorated ER stress by administering an oral treatment of TUDCA, a chemical chaperone^39^,^40^, from 17 to 22 wks on a HFD (Fig. 1). At 22 wks, the TUDCA treatment significantly improved the parameters of fatty liver (CR-Veh v.s. CR-TU), i.e. liver weight, the liver weight/body weight ratio, TG content, total TG amount (Fig. 5A–D), and some lipid profiles (Table 1A) concomitant with a significant decrease in F4/80-positive hepatic macrophage numbers (Fig. 6A–C), a slight decrease in the gene expression levels of TNF-α (Fig. 6D). TUDCA treatment significantly decreased the gene expression levels of XBP1s produced by mRNA splicing (CR-Veh v.s. CR-TU; Fig. 6F) and the protein expression levels of p-eIF2α (CR-Veh v.s. CR-TU; Fig. 6H) and CHOP (CR-Veh v.s. CR-TU; Fig. 6I), but not p-IRE1α (Fig. 6G), indicating the significant amelioration of ER stress. On the other hand, TUDCA treatments induced a slight paradoxical increase in the GRP78 protein expression, although statistically not significant (CR-Veh v.s. CR-TU; Fig. 6J). Since GRP78 protein inhibits hepatic lipogenesis^11^,^38^, the paradoxical slight increase in GRP78 protein expression might, at least partly, contribute to the amelioration of hepatic fat deposition by TUDCA treatment.

We summarized the changes in ER stress response in Supplemental Table S3. Consistent induction of protein expression levels of p-eIF2α and CHOP was observed both at 17 wks (Fig. 4H,I) (AD v.s. CR, cohort 2) and 22 wks (Fig. 6H,I) (AD-Veh v.s. CR-Veh, cohort 3), supporting an involvement of ER stress augmentation in the continuous deterioration of hepatic steatosis after undernourishment *in utero*. On the other hand, the gene splicing of XBP1u (XBP1s) (Fig. 4F) and protein expression of p-IRE1α (Fig. 4G) was transiently induced at 17 wks (cohort2), but not at 22 wks (Fig. 6F,G) (cohort 3); however, TUDCA treatment significantly suppressed gene splicing of XBP1u (XBP1s) only in CR pups (CR-Veh v.s. CR-TU), but not in AD pups (AD-Veh v.s. AD-TU) (Fig. 6F) (cohort 3), suggesting a potential involvement of XBP1s in the deterioration of hepatic steatosis between 17 wks and 22 wks. We have currently no clear explanation of the transient induction of p-IRE1α and its involvement in the continuous deterioration of hepatic steatosis between 17 wks and 22 wks. We speculated that heterogeneous factors might affect the complicated cascades of the p-IRE1α after their initial activation by the introduction of an obesogenic diet between 9 wks and 17 wks. Further studies are needed in order to confirm this speculation. Undernourishment *in utero* also caused consistent induction of the gene expression of endoplasmic reticulum–localized DnaJ 4 (ERdj4) and growth arrest and DNA damage inducible 34 (GADD34), but not activating transcription factor 4 (ATF4), at 17 wks (cohort 2; AD v.s. CR) and 22 wks (cohort 3; AD-Veh v.s. CR-Veh) (Supplemental Table S4). Taken together, ER stress responses of at least p-eIF2α, CHOP, ERdj4, GADD34, and possibly XBP1s were considered to be involved in the deterioration of hepatic steatosis between both 17 wks and 22 wks.

Therefore, significant induction of ER stress markers (AD v.s. CR; cohort 2) and specific alleviation of hepatic steatosis by TUDCA treatment (CR-Veh v.s. CR-TU; cohort 3) together supported the specific involvement of ER stress integration in the relationship between the predisposition of hepatic steatosis and undernourishment *in utero*.

Thus the results of present study lead us to an interesting conclusion that undernourishment *in utero* may program the future integration of ER stress and subsequent UPR in the liver on an obesogenic diet in later life and may, as a consequence, induce the deterioration of hepatic steatosis. However, the concept of long-lasting programing of ER stress response has not been established so far and little information is available concerning its mechanistic background, as far as we know. Intensive investigation is necessary to clarify the mechanism of long-lasting programing of ER stress response. The candidates of the future investigation would be the epigenetic changes of the key substances of ER stress response, such as XBP1u, IRE1α, eIF2α, CHOP, GRP78 etc. On the other hand, gut-liver axis was recently proposed as a causatively linking between NAFLD and bowel inflammation^41^. Gjymishka *et al.*, reported a possible involvement of ER stress in the pathogenesis of inflammatory bowel diseases^42^. Since we orally treated TUDCA, the alleviation of ER stress in bowel microbiota may be a candidate of the mechanism inducing the improvement of hepatic steatosis. Future investigation is necessary.

A limitation in the present study was the lack of the assessment of insulin sensitivity. There were no significant changes in the levels of casual blood glucose and insulin levels, although there was a slight decrease in blood glucose levels in CR-TU pups compared to CR-Veh (Table 1A). Fasting blood glucose and insulin levels as well as glucose tolerance testing are necessary to assess the involvement of the changes of insulin sensitivity in the deterioration of hepatic steatosis in this animal model. Another limitation is that we assessed the numbers of F4/80-positive hepatic macrophages but did not show any data of their characteristics, such as apoptosis and population. Rather stable numbers of leukocyte common antigen CD45-positive cells (Supplemental Table S5) suggested a possible changes in the leukocyte population; however, more intensive analysis of the changes in the entire immune system including F4/80-positive hepatic macrophages is necessary to clarify the detailed association between ER stress integration and chronic inflammation during the development of hepatic steatosis.

The results of the present study suggested that integration of the ER stress response was causatively associated with the developmental origins of NAFLD in individuals who were born small, even in developed countries^5^,^6^, and/or in those exposed to a low nutritional environment during fetal life due to maternal poverty or political turmoil, especially in developing countries such as China^2^. It may also be a concern after future economic and political reconstructions of current conflict areas such as the Middle East and Africa.

In Japan, large numbers of young women have a strong desire to be thin^43^, and insufficient energy intake by pregnant Japanese women^44^ has been implicated in the continuous decrease in mean birth weights as well as the continuous increase in the rate of low birth weight neonates in Japan^45^. The prevalence of undernourishment *in utero* may explain, at least partly, why the prevalence of NAFLD in Japan has nearly doubled in the last 10–15 years^2^.

The ER stress response recently emerged as a promising therapeutic target in metabolic syndrome including NAFLD by chemical chaperones^15^ or specific foods, such as Asian traditional brown rice^46^. The present study provides an insight into the development of strategies for early interventions in a potential high-risk population of NAFLD, such as people born small or those exposed to maternal undernourishment during the fetal period due to socioeconomic conditions^5^,^6^.

In conclusion, the results of the present study suggest that ER stress integration plays a pivotal role in priming the deterioration of hepatic steatosis by undernourishment *in utero*.

## Additional Information

**How to cite this article**: Muramatsu-Kato, K. *et al.* Undernourishment *in utero* Primes Hepatic Steatosis in Adult Mice Offspring on an Obesogenic Diet; Involvement of Endoplasmic Reticulum Stress. *Sci. Rep.* **5**, 16867; doi: 10.1038/srep16867 (2015).

## Supplementary Material

Supplementary Information

## Figures and Tables

**Table 1:** Effects of the TUDCA treatment on plasma lipid profiles as well as levels of transaminases and insulin (A), scores of steatosis and cell ballooning (B), and hydroxyproline content (C) (cohort 3).

A				
	AD-Veh (8)	AD-TU (10)	CR-Veh (7)	CR-TU (10)
Total cholesterol (mg/dl)	152.4 ± 39.4	137.1 ± 37.3	240.9 ± 39.1^#^	150.0 ± 40.9**
Chylomicron cholesterol (mg/dl)	1.0 ± 0.4	0.8 ± 0.3	0.7 ± 0.1	0.8 ± 0.4
VLDL cholesterol (mg/dl)	8.0 ± 3.2	4.7 ± 2.1	6.1 ± 2.2	5.0 ± 2.1
LDL cholesterol (mg/dl)	34.2 ± 10.8	31.2 ± 15.6	57.8 ± 19.0^#^	31.4 ± 9.4*
HDL cholesterol (mg/dl)	109.2 ± 31.6	100.5 ± 26.0	176.4 ± 18.2^#^	112.9 ± 33.8**
Small dense LDL cholesterol (mg/dl)	22.1 ± 11.3	21.2 ± 15.7	45.0 ± 15.5^#^	21.6 ± 10.1*
Total triglyceride (mg/dl)	74.4 ± 31.2	57.9 ± 18.6	42.0 ± 11.2	60.7 ± 25.4
AST (U/L)	136.4 ± 43.8	96.1 ± 48.6	146.7 ± 96.6	94.4 ± 46.3^#^
ALT (U/L)	44.4 ± 20.6	36.0 ± 24.5	87.0 ± 34.1^##^	46.3 ± 22.8
Glucose (mg/ml)	192.4 ± 37.0	180.6 ± 30.4	213.3 ± 44.2	187.1 ± 23.7
Insulin (ng/ml)	7.69 ± 1.09	6.00 ± 2.56	7.70 ± 0.70	8.19 ± 0.60
B				
	AD-Veh	AD-TU	CR-Veh	CR-TU
Steatosis (Grade)	2.33 ± 0.52 (6)	1.00 ± 1.10 (6)	3.00 ± 0.00^##^ (6)	1.5 ± 1.22* (6)
Cell ballooning	1.50 ± 0.55 (6)	0.83 ± 0.98 (6)	2.00 ± 0.00 (6)	1.00 ± 0.89 (6)
C				

Hydroxyproline content/total protein (μg/mg)	0.53 ± 0.10 (6)	0.82 ± 0.24 (6)	0.49 ± 0.09 (6)	0.71 ± 0.11 (6)

Data were expressed as the mean ± standard deviation.

^#^P < 0.05 v.s. AD-Veh. ^##^P < 0.05 v.s. AD-TU, *P < 0.05 v.s. CR-Veh, **P < 0.01 v.s. CR-Veh.
