# Supplementary material for: Undernourishment in utero Primes Hepatic Steatosis in Adult Mice Offspring on an Obesogenic Diet; Involvement of Endoplasmic Reticulum Stress
Source: Sci Rep. 2015 Nov 19;5:16867. doi: 10.1038/srep16867 (PMC4652266; doi:10.1038/srep16867)
Supplement: Supplementary Information [file srep16867-s1.doc]

**Undernourishment *in utero* Primes Hepatic Steatosis in Adult Mice Offspring on an Obesogenic Diet; Involvement of Endoplasmic Reticulum Stress**

Keiko Muramatsu-Kato^1^, Hiroaki Itoh*^,1^, Yukiko Kohmura-Kobayashi^1^, Urmi J Ferdous^1^, Naoaki Tamura^1^, Chizuko Yaguchi^1^, Toshiyuki Uchida^1^, Kazunao Suzuki^1^, Koshi Hashimoto^2^, Takayoshi Suganami^3,5^, Yoshihiro Ogawa^4,6^, and Naohiro Kanayama^1^

*^1^Department of Obstetrics and Gynecology, Hamamatsu University School of Medicine, Hamamatsu 431-3192 Japan.*

*^2^Department of Preemptive Medicine and Metabolism, ^3^Department of Organ Network and Metabolism, ^4^Department of Molecular Endocrinology and Metabolism, Graduate School of Medical and Dental Sciences, Tokyo Medical and Dental University, Tokyo 113-8510, Japan*

*^5^Japan Science and Technology Agency, PRESTO, Tokyo, Japan.*

*^6^Japan Science and Technology Agency, CREST, Tokyo, Japan.*

***Supplemental Figure S2; Bars of Western blot analysis (Fig. 2E-J; cohort 1, at 9 wks), (Fig. 3E, F, Fig. 4G-J; cohort 2, at 17 wks), and (Fig. 5I, J, Fig. 6G-J; at cohort 3, 22 wks).*** Experimental procedure was described in Figure 1. AD; Pups with normal fetal nourishment of maternal *ad libitum* feeding. CR; Pups with maternal caloric restriction. Veh; Vehicle. TU; Tauroursodeoxycholic acid. In cohort 2, squares with dotted line indicate blots of one identical specimen (duplicate, total two) as an internal positive control. In cohort 3, squares with dotted line indicate two identical specimens （in duplicate; total four）as internal positive controls.


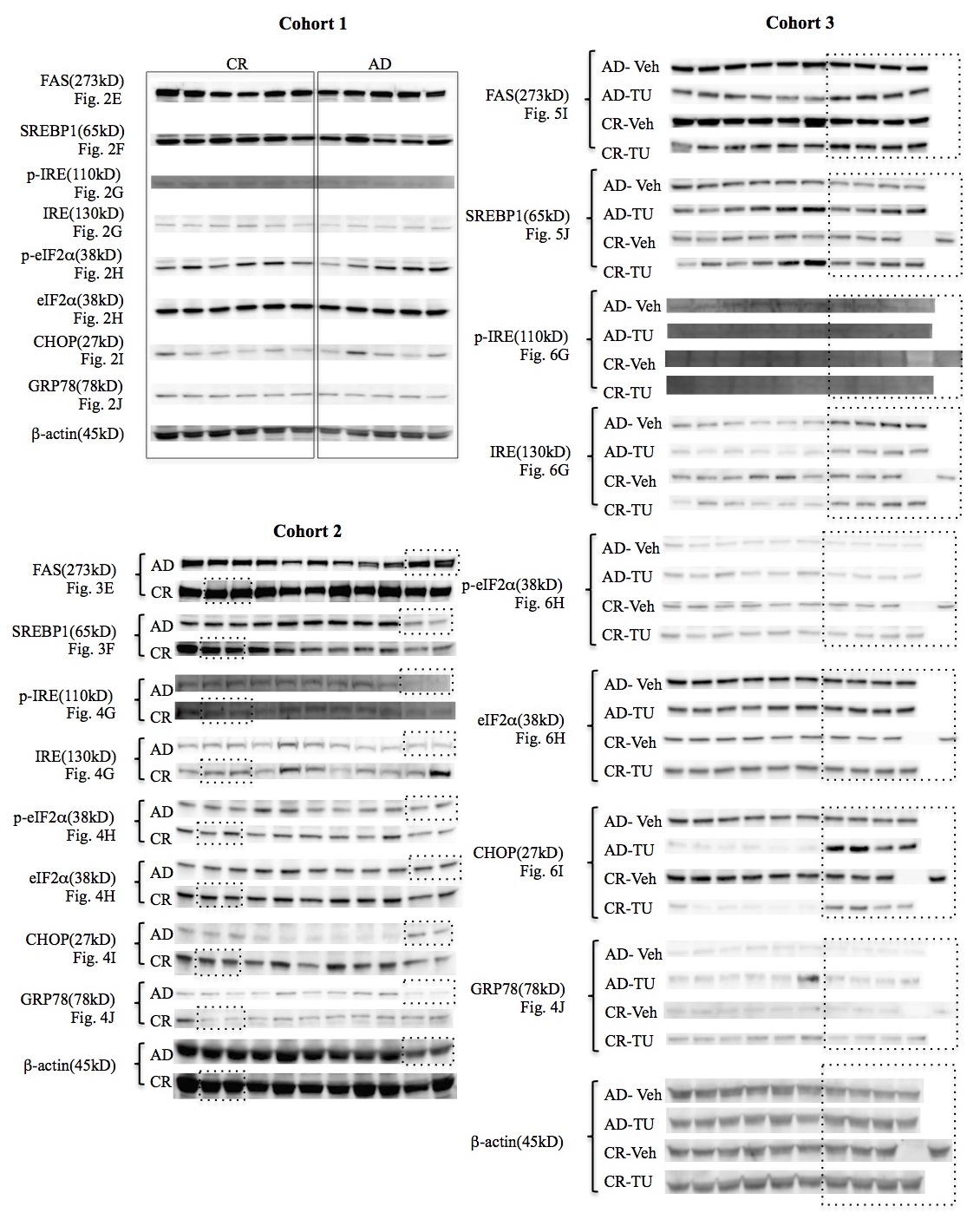


***Supplemental Figure S3; Representative Picrosirius Red stainings of the liver (cohort 3, 22 wks).*** Experimental procedure was described in Figure 1. AD; Pups with normal fetal nourishment of maternal *ad libitum* feeding. CR; Pups with maternal caloric restriction. Veh; Vehicle. TU; Tauroursodeoxycholic acid.

| Restriction of maternal caloric intake during pregnancy | LW (g) | | LW/ BW (%) | |
| --- | --- | --- | --- | --- |
|  | AD | CR | AD | CR |
| 70%  (30% reduction) | 1.8±0.63  (9) | 1.8±0.58  (27) | 4.54±1.06  (9) | 4.48±0.94  (27) |
| 65%  (35% reduction) | 1.8±0.46  (15) | 2.6±0.45 ***  (11) | 4.38±0.67  (15) | 5.02±1.71  (11) |
| 60%  (40% reduction) | 2.1±0.6  (36) | 2.4±0.56 *  (33) | 4.72±1.13  (36) | 5.31±1.07 *  (33) |
| 55%  (45% reduction) | 2.1±0.6  (38) | 2.0±0.51  (32) | 4.72±1.13  (38) | 4.69±0.92  (32) |

***Supplemental Table S1; Liver weight (LW) and the LW/ body weight (BW) ratio at 17 wks on a HFD in pups with different conditions of maternal caloric restriction (70-55%) during pregnancy.*** AD; Pups with normal fetal nourishment of maternal *ad libitum* feeding. CR; Pups with maternal caloric restriction. *; P<0.05, ***; P<0.001

A

|  | | AD | CR |
| --- | --- | --- | --- |
| Mean body weight (BW; g) | | 21.80±1.20  (21) | 21.86±1.35  (19) |
| Mean caloric intake | |  |  |
|  | Calorie/day | 0.47*  (21) | 0.53*  (19) |
|  | Mean caloric intake/mean BW (calorie/g) | 0.022*  (21) | 0.024*  (19) |

B

|  | | AD | CR |
| --- | --- | --- | --- |
| Mean body weight (BW; g) | | 44.03±3.08  (37) | 44.77±2.45  (33) |
| Mean caloric intake | |  |  |
|  | Calorie/day | 4.37±0.38  (37) | 4.34±0.39  (33) |
|  | Caloric intake/BW  (calorie/g) | 0.100±0.010  (37) | 0.097±0.011  (33) |

C

|  | | AD | | CR | |
| --- | --- | --- | --- | --- | --- |
|  | | Veh | TU | Veh | TU |
| Mean body weight (BW; g) | | 45.96±1.72  (8) | 43.44±2.85  (10) | 45.46±1.17  (7) | 44.54±2.50  (10) |
| Mean caloric intake | |  |  |  |  |
|  | Calorie/day | 5.49±0.33  (8) | 5.23±0.51  (10) | 5.46±0.15  (7) | 5.53±0.40  (10) |
|  | Caloric intake/BW (calorie/g) | 0.120±0.010  (8) | 0.121±0.013  (10) | 0.120±0.004  (7) | 0.124±0.007  (10) |

***Supplemental Table S2; Mean body weight and mean caloric intake of cohort 1 (A; 9 wks), cohort 2 (B; 17 wks), and cohort 3 (C; 22 wks).*** Experimental procedure of each cohort were described in Figure 1. *; Mean caloric intake was estimated by total caloric intake of each cage for 4 or 5 pups. AD; Pups with normal fetal nourishment of maternal *ad libitum* feeding. CR; Pups with maternal caloric restriction. Veh; Vehicle. TU; Tauroursodeoxycholic acid.

**A**

|  | XBP1s/XBP1u | p-IRE1α/IRE1α | p-eIF2α/eIF2α | CHOP/β-actin | GRP78/β-actin  (Inhibiting hepatic lipogenesis)  Reference 38 |
| --- | --- | --- | --- | --- | --- |
| 17 wks | AD<CR  P<0.05  (Fig. 4F) | AD<CR  P<0.05  (Fig. 4G) | AD<CR  P<0.01  (Fig. 4H) | AD<CR  P<0.001  (Fig. 4I) | AD~CR  NS  (Fig. 4J) |
| 22 wks | AD-Veh~CR-Veh  NS  (Fig. 6F) | AD-Veh>CR-Veh  P<0.05  (Fig. 6G) | AD-Veh<CR-Veh  P<0.05  (Fig. 6H) | AD-Veh<CR-Veh  P<0.05  (Fig. 6I) | AD-Veh~CR-Veh  NS  (Fig. 6J) |

**B**

|  | XBP1s/XBP1u | p-IRE1α/IRE1α | p-eIF2α/eIF2α | CHOP/β-actin | GRP78/β-actin  (Inhibiting hepatic lipogenesis)  Reference 38 |
| --- | --- | --- | --- | --- | --- |
| 22 wks | CR-Veh>CR-TU  P<0.05  (Fig. 6F) | CR-Veh~CR-TU  NS  (Fig. 6G) | CR-Veh>CR-TU  P<0.05  (Fig. 6H) | CR-Veh>CR-TU  P<0.05  (Fig. 6I) | CR-Veh~CR-TU  NS  (Fig. 6J) |

***Supplemental Table S3; Summary of the changes in ER stress responses observed in CR pups (AD v.s. CR at 17 wks [cohort 2] and AD-Veh v.s. CR-Veh at 22 wks [cohort 3])(A) and those with TUDCA s (CR-Veh v.s. CR-TU at 22 wks [cohort 3])(B) described in Fig 4 and Fig 6.*** Experimental procedures were described in Figure 1. AD; Pups with normal fetal nourishment of maternal *ad libitum* feeding. CR; Pups with maternal caloric restriction. Veh; Vehicle. TU; Tauroursodeoxycholic acid. NS; No significant difference.

|  | ATF4 | | ERdj4 | | GADD34 | |
| --- | --- | --- | --- | --- | --- | --- |
| 17 wks  cohort 2 | AD  1.91±0.63  (AU)  (9) | CR  1.94±0.86  (AU)  (9) | AD  0.62±0.16  (AU)  (9) | CR  0.92±0.28^#^  (AU)  (9) | AD  7.24±1.81  (AU)  (9) | CR  10.11±2.77^#^  (AU)  (9) |
| 22 wks  cohort 3 | AD-Veh  1.60±0.57  (AU)  (6) | CR-Veh  1.81±0.66  (AU)  (6) | AD-Veh  0.86±0.17  (AU)  (6) | CR-Veh  1.26±0.32*  (AU)  (6) | AD-Veh  8.45±0.94  (AU)  (6) | CR-Veh  10.16±1.77*  (AU)  (6) |

***Supplemental Table S4; Gene expression of activating transcription factor 4 (ATF4), endoplasmic reticulum–localized DnaJ 4 (ERdj4) and growth arrest and DNA damage inducible 34 (GADD34).*** Experimental procedures were described in Figure 1. AD; Pups with normal fetal nourishment of maternal *ad libitum* feeding. CR; Pups with maternal caloric restriction. Veh; Vehicle. TU; Tauroursodeoxycholic acid. AU; arbitrary unit. Data were expressed as the mean ± standard deviation. ^#^; P<0.05 v.s. AD. *; P<0.05 v.s. AD-Veh, The gene expression level of each gene was determined by quantitative RT-PCR using the High Capacity RNA to cDNA Master Mix (Applied Biosystems, Foster City, CA) and SYBR Green PCR Master Mix (Applied Biosystems). The expression of 18S ribosomal RNA was used as an internal control. The primers used were ATF4; forward; GAGTTTAGCAAAGCAAGGGG, reverse; AACAGCAGCCACACTGCTAC, ERdj4; forward; CCCCAGTGTCAAACTGTACCAG, reverse; AGCGTTTCCAATTTTCCATAAATT, GADD34; forward; CTTCGCGAGCAGTCCGGA, reverse;　GACAGGAGATAGAAGTTGTGG, and 18S ribosomal RNA; forward; GGGAGCCTGAGAAACGGC, reverse; GGGTCGGGAGTGGGTAATTTT.

A

|  | AD | CR |
| --- | --- | --- |
| Average number of CD45  positive cells/HPF | 5.04±1.01  (9) | 4.68±1.77  (10) |

***B***

|  | AD | | CR | |
| --- | --- | --- | --- | --- |
|  | Veh | TU | Veh | TU |
| Average number of CD45 positive cells/HPF | 8.04±1.51  (6) | 7.56±2.00  (6) | 6.83±1.64  (6) | 6.79±1.88  (6) |

***Supplemental Table S5; Average number of CD45 positive cells/HPF of cohort 2 (A; 17 wks) and cohort 3 (B; 22 wks).*** Experimental procedures were described in Figure 1. AD; Pups with normal fetal nourishment of maternal *ad libitum* feeding. CR; Pups with maternal caloric restriction. Veh; Vehicle. TU; Tauroursodeoxycholic acid.
